# Effects of dietary taurine supplementation on polyunsaturated fatty acids, cholesterol, and egg quality of egg of hens

**DOI:** 10.3389/fnut.2026.1746365

**Published:** 2026-03-12

**Authors:** Shunyang Wang, Qiqi Zhang, Huimin Lu, Meng Yu, Mahoud M. Alagawany, Cheng Chen, Zhongxin Zhou

**Affiliations:** 1 Department of Animal Nutrition and Feed Science, College of Animal Sciences & Technology, Huazhong Agricultural University, Wuhan, China; 2 Poultry Department, Faculty of Agriculture, Zagazig University, Zagazig, Egypt; 3 College of Animal Science and Technology, Shihezi University, Shihezi, China

**Keywords:** cholesterol, egg quality, laying hens, polyunsaturated fatty acids, taurine

## Abstract

**Objective:**

This study aimed to assess the impact of dietary taurine supplementation on egg quality, polyunsaturated fatty acids, and cholesterol contents in egg during the late laying period in hens.

**Methods:**

A total of 102 44-week-old Roman pink laying hens were randomly divided into six groups, one control group and five treatment groups additionally supplied with 0.01, 0.02, 0.05, 0.1, and 0.2% Taurine (weight/weight). Feed intake and body weight were recorded to calculate production performance, and eggs were collected to analyze egg quality. At the end of the study, 10 laying hens were randomly selected from each group and slaughtered to collect blood, liver, and other samples.

**Results:**

The results revealed that 0.05% or 0.1% taurine significantly increased the contents of C18-3n and C18-2n polyunsaturated fatty acids in yolks (*p* < 0.05). Dietary 0.1% or 0.2% taurine significantly decreased the total cholesterol content (*p* < 0.05) in egg yolks. Moreover, 0.05, 0.1, and 0.2% taurine significantly reduced the mRNA expression of the cholesterol synthesis rate-limiting enzyme *HMGCR* and transcription factor *SREBP2* in the livers of aged laying hens (*p* < 0.05), and promoted hepatic cholesterol transporter protein *ABCG5* and cholesterol esterase *ACAT2* mRNA expression (*p* < 0.05); 0.2% taurine promoted hepatic bile acid synthesis rate-limiting enzyme *CYP7A1* mRNA expression (*p* < 0.05). Additionally, supplementation with 0.01 to 0.2% taurine significantly increased egg weight, with 0.2% taurine significantly increased egg Albumen height at week 4, with 0.05 and 0.1% taurine significantly increased egg Haugh units at week 12, and with 0.02 to 0.2% taurine decreased yolk color at week 16 (*p* < 0.05).

**Conclusion:**

Supplying 0.05 to 0.2% taurine improved specific aspects of egg quality, enhanced contents of polyunsaturated fatty acids, and reduced egg yolk cholesterol during the late laying period. This study provides scientific evidence for the further taurine use in egg production.

## Graphical abstract

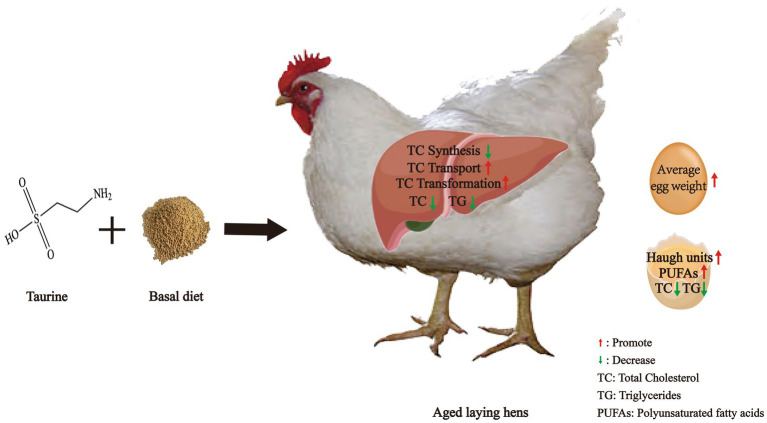

## 1 Introduction

Laying hens enter the late laying period at approximately 45 weeks of age. The “extended breeding” strategy in the laying hen industry has prolonged this period, leading to economic losses from reduced productivity and egg quality, as well as welfare concerns such as increased susceptibility to metabolic disorders (1, 2). During this phase, the laying rate and egg quality decline during this time, indicated by indices such as Albumen height, Haugh unit reduction, and increased total cholesterol (TC) in the egg (3–5). Therefore, improving egg quality in the late laying stage is being increasingly crucial in hens breeding industry.

Taurine is a sulfur-containing amino acid that widely exists in animals with highly abundant in vital organs such as the brain, heart, skeletal muscle, and liver (6–9). Taurine plays crucial physiological roles in regulating lipid metabolism (7, 10) and has anticancer (11), antioxidative, and antiaging effects (12). Owing to diverse physiological functions, taurine addition to feed has attracted much attention in poultry nutrition (13). Supplementing taurine for laying hens can reduce inflammation, increase their antioxidant capacities (14, 15), reduce kidney damage (16), increase eggshell quality during the late laying period (17, 18), effectively alleviate fatty liver hemorrhagic syndrome (19) and nonalcoholic fatty liver (20) in late laying hens. However, its effects on egg nutritional composition, particularly polyunsaturated fatty acids (PUFAs) and cholesterol, remain uncertain in aging hens, where age-related declines in lipid metabolism efficiency may alter nutrient deposition (21).

The dry matter of eggs contains approximately 52.7% protein and 39.7% fat (22). The yolk is particularly high in fat and contains more than 60% dry matter (23–25). Egg yolks contain PUFAs, such as omega-3 and omega-6 fatty acids, and serve as an important source of PUFAs for humans. These PUFAs are crucial for safeguarding cardiovascular health, promoting brain development, enhancing visual health, and exerting anti-inflammatory effects (26–28). These benefits are nutritionally meaningful, as modest increases in egg PUFA content can contribute to daily intake recommendations. Moreover, the yolk is abundant in triglycerides (TG) and TC. A medium-sized egg contains approximately 200 ~ 300 mg of TC (29, 30), classifying it as a high-TC food (31, 32).

The egg TC synthesis primarily occurs in the liver, TC homeostasis in the liver is maintained through the dynamic equilibrium among TC synthesis, transport, and transformation (33, 34). In mammals, Taurine suppresses hepatic TC synthesis by downregulating sterol regulatory element binding protein 2 (SREBP-2) and 3-hydroxy-3-methylglutaryl coenzyme A reductase (HMGCR) expression in high-fat diet-fed rats (35). In high-cholesterol diet-fed mouse, taurine promotes TC clearance by upregulating low-density lipoprotein receptor (LDLR) and enhancing cholesterol 7α-hydroxylase (CYP7A1) activity to accelerate conversion to bile acids (36). Taurine also facilitates hepatic TC efflux and storage by promoting ATP-binding cassette subfamily G member 5/8 (ABCG5/8)-mediated biliary excretion (37, 38) and increasing acetyl coenzyme A acyltransferase 2 (ACAT2)-mediated cholesterol esterification (39). In poultry, lipid metabolism differs, potentially linking age-related enzyme declines to reduced PUFA deposition and elevated TC. Taurine’s role in avian hepatic pathways may thus address these age-specific changes, but avian-specific data are limited.

Thus, this study aimed to investigate the impact of dietary taurine on laying hen performance, egg quality, and liver TC metabolism during the late laying period and provide a scientific basis for the application of taurine in poultry nutrition.

## 2 Materials and methods

### 2.1 Birds, diets, and management

The animal testing protocol for this study was approved by the Animal Protection and Use Committee of Huazhong Agricultural University (Approval ID: HZAUCH-2022–0020), and the animal experiments were performed at the experimental chicken farm of Huazhong Agricultural University. For the experiment, 102 healthy 44-week-old Roman pink laying hens with similar body weights and laying rates of approximately 93% were selected from the Hubei Academy of Agricultural Sciences. We randomly divided the laying hens into six groups of 17 birds each, following the principle of random allocation. We formulated a corn–soybean basal diet for the CON group based on The Chinese Chicken Feeding Standard (2004), while the five treatment groups received a basal diet containing 0.01, 0.02, 0.05, 0.1%, or 0.2% taurine (Table 1) (99.3% purity; Qianjiang Yongan Pharmaceutical Co., Ltd., China). Dosages were selected based on prior dose–response studies in poultry showing benefits at 0.05–0.2% without toxicity (18, 20). During the experiment, Hens were housed in individual metal cages (70 × 70 × 50 cm) at 16–24 °C, 40–60% humidity, 10–20 lux, and 16 h light/day. Water was ad libitum; feed was restricted to 110 g/day to simulate commercial practices preventing obesity in late-laying hens, though actual intake averaged slightly higher due to minor spillage/adjustments (Table 2). The trial lasted 18 weeks (2-week adaptation + 16-week formal).

**Table 1:** Basal diet formulation and nutrient levels.

Dietary ingredient	Supplemental levels of taurine (%)					
	0	0.01%	0.02%	0.05%	0.10%	0.20%
Corn	63.00	63.00	63.00	63.00	63.00	63.00
Soybean meal	24.00	24.00	24.00	24.00	24.00	24.00
Soybean Oil	0.50	0.50	0.50	0.50	0.50	0.50
Stone aggregates	6.50	6.50	6.50	6.50	6.50	6.50
Limestone	3.00	3.00	3.00	3.00	3.00	3.00
Premix^1^	3.00	3.00	3.00	3.00	3.00	3.00
Total	100	100	100	100	100	100
Taurine (99.99%)	-	0.01%	0.02%	0.05%	0.10%	0.20%
Nutrient levels						
Metabolic energy (MJ/kg)	11.06	11.06	11.06	11.06	11.06	11.06
Crude Protein (%)	15.38	15.38	15.38	15.38	15.38	15.38
Calcium (%)	3.53	3.53	3.53	3.53	3.53	3.53
Total Phosphorus (%)	0.48	0.48	0.48	0.48	0.48	0.48
Non-phytate phosphorus	0.28	0.28	0.28	0.28	0.28	0.28
Lysine	0.77	0.77	0.77	0.77	0.77	0.77
Methionine	0.38	0.38	0.38	0.38	0.38	0.38
Methionine + Cystine	0.63	0.63	0.63	0.63	0.63	0.63

^1^Premix provided per kilogram of diet: vitamin A 8000 IU, vitamin D3 3,300 IU, vitamin E 25 IU, vitamin K 2.75 mg, vitamin B1 2.75 mg, vitamin B2 6.00 mg, vitamin B6 5.00 mg, vitamin B12 0.03 mg, biotin 0.105 mg, folic acid 1.00 mg, niacinamide 35 mg, pantothenic acid 10 mg, Mn 100 mg, Zn 85 mg, Fe 60 mg, Cu 10 mg, I 1 mg, Se 0.3 mg, Co 0.2 mg.

**Table 2:** Primers used for qPCR.

Gene	Gen Bank ID	Primer sequence (5′ to 3′)	Products (bp)
*HMGCR*	NM_204485.3	F: GCAGATGGGATGACTCGAGG R: TAGGCGGGCAAACCTACTTG	141
*SREBP-2*	XM_040660556.2	F: CCCAGAACAGCAAGCAAGG R: GCGAGGACAGGAAAGAGTG	108
*CYP7A1*	NM_001001753.2	F: TGGTAGCATTGACCCAGCAG R: TCTTGACTGCAGCATGACGT	139
*ABCG5*	XM_419457.8	F: GTGGACACTCGAAGCAAGGA R: TGAACGGTATGGGTGGAAGC	151
*LDLR*	NM_204452.1	F: CCACCATTTGGCAGAGGAA R: ACCGCAGTCAGACCAGAAGAG	86
*ACAT2*	NM_001039287.3	F: CCTCGACATGGGAGCAACTT R: CCCGTCTGAGCCCAAGATAC	197
*β-actin*	NM_205518.2	F: ATCCGGACCCTCCATTGTC R: AGCCATGCCAATCTCGTCTT	120

### 2.2 Sample collection

During the experimental period, the number of eggs and egg weights were determined daily (9:00 a.m.) according to groups. The laying rates were calculated, and the food intake per chicken was recorded. The average egg weight was the average weight of all eggs laid per bird, the average daily feed intake = feed intake (g)/time (d), and the feed-to-egg ratio = feed intake/ (egg weight*egg production). At weeks 4, 8, 12 and 16 of the experiment, 10 eggs were randomly selected from each group of hens for egg quality determination. At the end of the experiment (62 weeks), 10 laying hens were randomly selected from each group, and blood was collected from the maxillary vein; the blood was centrifuged at 3000 r/min for 10 min at 4 °C, divided, and stored at −80 °C for serum TG and TC determination. Following blood collection, the hens were humanely euthanized by rapid cervical dislocation, to ensure immediate unconsciousness and minimize suffering, and the livers were removed, snap-frozen in liquid nitrogen, and stored at −80 °C for subsequent gene expression analysis.

### 2.3 Measurement of egg quality

We used an eggshell strength tester (Model KQ-1A, Tenovo Food, Beijing, China) to determine eggshell strength. Eggshell thickness was measured as the average eggshell thickness across three points: the blunt end, the sharp end, and the middle portion of the egg. The egg shape index was measured as the longitudinal diameter/transverse diameter. Yolk color was assessed via a Roche colorimetric fan. The Albumen height and Haugh units were determined via a multifunctional egg quality analyzer (EMT-5200, Robotmation Co., Ltd., Tokyo, Japan).

### 2.4 Nutritional assessment of eggs

At the end of the experiment (62 weeks), 8 eggs were randomly selected from each group, and their dry matter, crude fat, crude ash, and crude protein contents were determined. According to the manufacturer’s instructions, the TC and TG contents in the egg yolks were determined via analysis kits (Nanjing Jianjian Bioengineering Institute, Nanjing, China).

### 2.5 Measurement of the antioxidant capacities of egg yolks

At the end of the experiment (62 weeks), 6 eggs were randomly selected from each group, and the total superoxide dismutase (T-SOD) activity, total antioxidant capacity (T-AOC), and malondialdehyde (MDA) content of each yolk were determined via analysis kits (Nanjing Jianjian Bioengineering Institute, Nanjing, China).

### 2.6 Measurement of fatty acids in egg yolk

At the end of the experiment (62 weeks), we randomly selected 3 eggs each from the CON group and the 0.05, 0.1, and 0.2% taurine-treated groups. We then separated the yolks and placed them in a 25 L vacuum freeze drier (Genesis SQ Super ES-55, VirTis, Warminster, PA, USA) for 72 h. The yolks were then ground into powder, and the fatty acids were extracted, and analyzed via GC–MS (Model 8,890-5977B, Agilent Technologies, Santa Clara, CA, USA) to determine the fatty acid composition and contents.

### 2.7 Serum and liver biochemical analysis

The TG and total TC levels in the livers and serum were determined via analysis kits (Nanjing Jiancheng Biological Engineering Institute, Nanjing, China) according to the manufacturer’s instructions.

### 2.8 RNA extraction and quantitative real-time PCR

In accordance with the manufacturer’s instructions, TRIZOL reagent (Invitrogen, Waltham, MA) was added to liver samples, and RNA was extracted by adding chloroform. RNA was precipitated by adding precooled isopropanol, washed with 75% ethanol, and the precipitate was then solubilized in DEPC water. The RNA concentration was determined via an ultramicro spectrophotometer (Nanodrop 2000, Thermo Scientific, Waltham, MA, USA). cDNA was synthesized via TRUEscript RT Master Mix (Adderall Biotechnology Ltd., Beijing, China). Primers for key genes involved in TC metabolism were designed via Primer 6.0 software (Table 3). A real-time fluorescence quantitative PCR assay was performed to determine the expression of *HMGCR*, *SREBP-2*, *CYP7A1*, *ABCG5*, *LDLR*, and *ACAT2* using Bestar® SYBR Green qPCR Master Mix (Shanghai Xinghan Biotechnology Co., Ltd., Shanghai, China) according to the manufacturer’s instructions. *β*-actin was used as an internal reference gene, and the relative expression was calculated according to the 2^-ΔΔCT^ method. Three parallel qPCRs were performed for each sample.

**Table 3:** Effects of taurine on the production performance of laying hens during the late laying period.

Item	Treatment time	CON	+ 0.01% Tau	+ 0.02% Tau	+ 0.05% Tau	+ 0.1% Tau	+ 0.2% Tau	*p* value	Linear	Quadratic
Laying rate (%)	3–8 weeks of age	88.84 ± 4.49	88.73 ± 6.27	88.24 ± 8.05	91.18 ± 6.29	89.43 ± 6.68	92.86 ± 2.70	0.249	0.054	0.106
	9–16 weeks of age	83.93 ± 8.22	88.33 ± 6.98	86.62 ± 9.69	87.64 ± 13.24	88.43 ± 5.68	89.88 ± 5.43	0.517	0.089	0.232
Average daily feed intake (g/d)	3–8 weeks of age	114.93 ± 0.28	114.92 ± 0.20	114.95 ± 0.21	114.95 ± 0.23	114.87 ± 0.28	114.91 ± 0.26	0.942	0.625	0.840
	9–16 weeks of age	114.65 ± 0.24	114.70 ± 0.23	114.62 ± 0.27	114.66 ± 0.24	114.65 ± 0.29	114.67 ± 0.22	0.972	0.911	0.950
Average egg weight (g)	3–8 weeks of age	56.80 ± 0.42	57.99 ± 0.32*	57.73 ± 0.37*	58.09 ± 0.54*	58.42 ± 0.34*	58.86 ± 0.36*	**< 0.001**	**< 0.001**	**< 0.001**
	9–16 weeks of age	56.13 ± 0.23	56.91 ± 0.55*	57.86 ± 0.41*	58.22 ± 0.23*	58.55 ± 0.40*	58.65 ± 0.20*	**< 0.001**	**< 0.001**	**< 0.001**
Feed-to-egg ratio	3–8 weeks of age	2.28 ± 0.12	2.24 ± 0.17	2.28 ± 0.23	2.25 ± 0.39	2.21 ± 0.17	2.10 ± 0.06	0.243	**0.033**	**0.047**
	9–16 weeks of age	2.46 ± 0.27	2.30 ± 0.20	2.32 ± 0.32	2.35 ± 0.69	2.23 ± 0.14	2.19 ± 0.14	0.348	**0.040**	0.123

The values represent the mean ± SD (*n* = 10), *p* < 0.05 was considered to indicate statistical significance, and values in the same row without same superscript letter indicate significant difference (*p* < 0.05). *p < 0.05 between CON and other groups.

### 2.9 Statistical analysis

The experimental data were analyzed via SPSS software (version 23.0; SPSS Inc., Chicago, IL, USA). t tests and one-way analysis of variance (ANOVA) were performed to analyze group differences. The data are presented as the mean ± standard deviation (SD); *p* < 0.05 was considered to indicate a statistically significant difference.

## 3 Results

### 3.1 Taurine improves the production performance of hens in the late laying period

The addition of 0.01, 0.02, 0.05, 0.1, and 0.2% taurine to the diets of the laying hens significantly increased the mean egg weights at weeks 3–8 and 9–16 (*p* < 0.01, Table 2) compared with those of the CON group. The laying rate showed no significant differences but exhibited near-significant linear increases (*p* = 0.054 and *p* = 0.089), with an average increase of about 4–5% compared to the CON group. The addition of taurine did not significantly affect average feed intakes, and feed-to-egg ratio (*p* > 0.05). But, compared with those in the CON group, the feed-to-egg ratios at weeks 3–8 and 9–16 decreased linearly (*p* < 0.05) with increasing taurine addition.

### 3.2 Taurine promotes egg quality in the late laying period

The study revealed no significant differences in eggshell strength, eggshell thickness, or egg shape index when 0.01, 0.02, 0.05, 0.1%, or 0.2% taurine was added to the diets compared with those of the CON group (*p* > 0.05, Table 4). Yolk color was significantly lower in eggs laid by hens fed diets enriched with 0.2% taurine at week 12 than in those from the CON group (*p* < 0.05); egg yolk color was significantly lower in eggs laid by hens fed with a taurine-supplemented diet at week 16 (*p* < 0.01), except in the 0.01% taurine group; and yolk color decreased linearly and quadratically with increasing dietary taurine percentages at both week 12 and week 16 (*p* < 0.001). Compared with that of the CON group, the Albumen height of the eggs laid by the 0.2% taurine-treated group at week 4 was significantly lower (*p* < 0.01) and decreased linearly and quadratically with increasing dietary taurine addition (*p* < 0.01); the Albumen height at weeks 8 and 12 showed linear and quadratic increases (*p* < 0.01), respectively; at week 16, an increase in taurine supplementation was associated with significant linear and quadratic effects on egg Albumen height (*p* < 0.01). Feeding hens diets containing 0.05 and 0.1% taurine significantly increased egg Haugh units (*p* < 0.05) and led to a quadratic increase (*p* < 0.01) at week 12 of rearing; feeding hens diets containing 0.05% taurine also significantly (*p* < 0.01) increased egg Haugh units at week 16. Interestingly, compared with the control diet, the taurine-supplemented diet did not increase egg Haugh unit at weeks 4 and 8 but did result in a linear increase (*p* < 0.05).

**Table 4:** Effects of taurine on the egg qualities of laying hens during the late laying period.

Item	Time	CON	+ 0.01% Tau	+ 0.02% Tau	+ 0.05% Tau	+ 0.1% Tau	+ 0.2% Tau	*p* value	Linear	Quadratic
Eggshell strength (N)	4 weeks of age	50.30 ± 2.24	48.44 ± 13.13	55.46 ± 7.92	48.47 ± 5.83	54.45 ± 4.88	52.99 ± 4.02	0.194	0.323	0.555
	8 weeks of age	57.45 ± 5.73	51.37 ± 6.55	51.46 ± 8.79	53.69 ± 3.20	53.38 ± 6.44	53.84 ± 7.29	0.380	0.956	0.813
	12 weeks of age	48.39 ± 3.15	50.28 ± 3.93	50.14 ± 4.60	51.49 ± 7.12	51.12 ± 4.64	49.88 ± 4.73	0.774	0.740	0.396
	16 weeks of age	44.75 ± 11.18	54.01 ± 4.67	52.19 ± 6.26	46.85 ± 5.13	41.47 ± 13.45	47.89 ± 13.24	0.080	0.370	0.247
Eggshell thickness (mm)	4 weeks of age	0.35 ± 0.02	0.37 ± 0.01	0.36 ± 0.02	0.35 ± 0.02	0.36 ± 0.02	0.36 ± 0.01	0.097	0.468	0.710
	8 weeks of age	0.34 ± 0.01	0.34 ± 0.02	0.34 ± 0.01	0.35 ± 0.01	0.36 ± 0.01	0.35 ± 0.02	0.072	**0.014**	**0.008**
	12 weeks of age	0.36 ± 0.01	0.36 ± 0.01	0.36 ± 0.00	0.36 ± 0.00	0.35 ± 0.01	0.36 ± 0.01	0.186	0.502	0.540
	16 weeks of age	0.32 ± 0.01	0.33 ± 0.02	0.33 ± 0.01	0.33 ± 0.01	0.33 ± 0.01	0.32 ± 0.01	0.207	0.263	0.139
Egg shape index	4 weeks of age	1.28 ± 0.03	1.29 ± 0.05	1.29 ± 0.02	1.27 ± 0.05	1.29 ± 0.04	1.31 ± 0.05	0.593	0.160	0.242
	8 weeks of age	1.34 ± 0.02	1.32 ± 0.05	1.30 ± 0.05	1.36 ± 0.04	1.35 ± 0.05	1.34 ± 0.04	0.053	0.166	0.171
	12 weeks of age	1.32 ± 0.03	1.31 ± 0.02	1.31 ± 0.05	1.29 ± 0.05	1.34 ± 0.02	1.33 ± 0.03	0.052	0.208	0.366
	16 weeks of age	1.32 ± 0.04	1.31 ± 0.02	1.29 ± 0.06	1.30 ± 0.05	1.34 ± 0.03	1.34 ± 0.04	0.052	**0.015**	0.055
Yolk color	4 weeks of age	10.32 ± 1.57	10.59 ± 2.18	11.40 ± 0.76	11.36 ± 3.44	10.55 ± 1.80	8.84 ± 1.12	0.072	**0.016**	**0.012**
	8 weeks of age	9.41 ± 0.24	9.12 ± 0.54	9.36 ± 4.33	9.12 ± 0.47	9.08 ± 0.30	8.89 ± 0.41	0.117	**0.012**	**0.040**
	12 weeks of age	9.16 ± 0.17	8.98 ± 0.42	9.00 ± 0.27	9.05 ± 0.31	8.72 ± 0.52	8.50 ± 0.47*	**0.004**	**< 0.001**	**< 0.001**
	16 weeks of age	8.79 ± 0.29	8.65 ± 0.62	7.98 ± 0.67*	8.08 ± 0.36*	8.27 ± 0.57*	7.60 ± 0.46*	**< 0.001**	**< 0.001**	**0.001**
Albumen height (mm)	4 weeks of age	6.39 ± 0.60	6.88 ± 1.48	6.72 ± 0.64	7.67 ± 1.20	6.17 ± 1.11	5.64 ± 0.89*	**0.002**	**0.006**	**0.007**
	8 weeks of age	6.33 ± 1.56	6.59 ± 1.63	7.44 ± 1.18	6.71 ± 0.55	7.14 ± 0.97	7.59 ± 1.24	0.167	**0.048**	0.141
	12 weeks of age	5.66 ± 1.97	6.86 ± 0.98	6.73 ± 0.67	7.11 ± 0.50	7.11 ± 0.30	6.33 ± 1.50	0.066	0.835	**0.040**
	16 weeks of age	5.97 ± 1.05	7.27 ± 0.83	7.11 ± 1.40	6.45 ± 2.47	6.67 ± 0.86	5.32 ± 1.71	0.064	**0.029**	**0.041**
Haugh units	4 weeks of age	80.42 ± 7.40	80.97 ± 9.44	79.63 ± 4.47	85.02 ± 6.49	76.19 ± 7.95	73.82 ± 5.89	**0.022**	**0.008**	**0.025**
	8 weeks of age	80.74 ± 8.29	83.40 ± 5.31	83.34 ± 7.00	78.34 ± 5.60	83.06 ± 6.52	87.36 ± 4.90	0.081	**0.040**	0.051
	12 weeks of age	75.35 ± 6.73	80.99 ± 6.39	80.42 ± 4.88	85.18 ± 2.06*	83.20 ± 1.70*	75.96 ± 13.18	**0.034**	0.488	**0.008**
	16 weeks of age	74.99 ± 7.67	83.66 ± 2.75	81.82 ± 8.97	83.90 ± 3.23*	80.03 ± 6.36	75.29 ± 6.34	**0.007**	0.135	**0.026**

The values represent the mean ± SD (*n* = 10), *p* < 0.05 indicates statistical significance, and values in the same row without the same superscript letter indicate significant differences (*p* < 0.05). *p < 0.05 between the CON group and the other groups.

### 3.3 Taurine decreases the TG and TC contents in egg yolks

**Figure 1:**
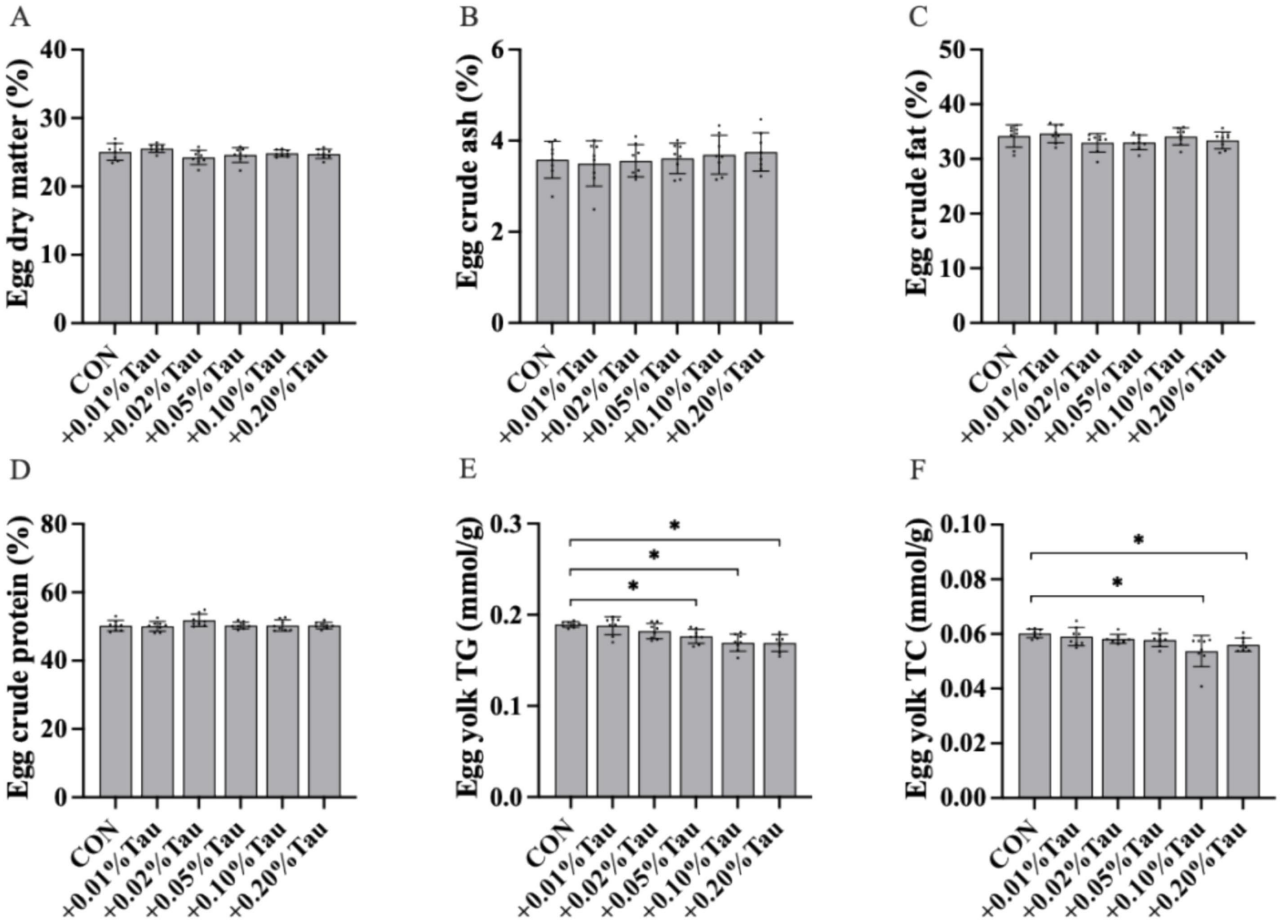
Effects of taurine on the nutritional level of eggs. **(A)** Moisture contents of eggs. **(B)** Crude ash contents of eggs. **(C)** Crude fat contents of eggs. **(D)** Crude protein contents in eggs. **(E)** TG contents in eggs. **(F)** TC contents in eggs. Results regarding other components are based on the results of dry matter determination; the values represent the mean ± SD (*n* = 8), *p < 0.05 between the CON group and the other groups.

Compared with the CON diet, the diets supplemented with 0.01, 0.02, 0.05, 0.1%, or 0.2% taurine did not significantly affect dry matter, crude ash, crude fat, or crude protein in eggs (*p* > 0.05; Figures 1A–D). The amount of TG in egg yolks was significantly lower in the groups that were given 0.05, 0.1%, or 0.2% taurine than in the CON group (*p* < 0.05; Figure 1E). The amount of TC in egg yolks was also significantly lower in the 0.1 and 0.2% taurine-treated groups than in the CON group (*p* < 0.05; Figure 1F).

### 3.4 Taurine increases the antioxidant capacities of egg yolks

**Figure 2:**
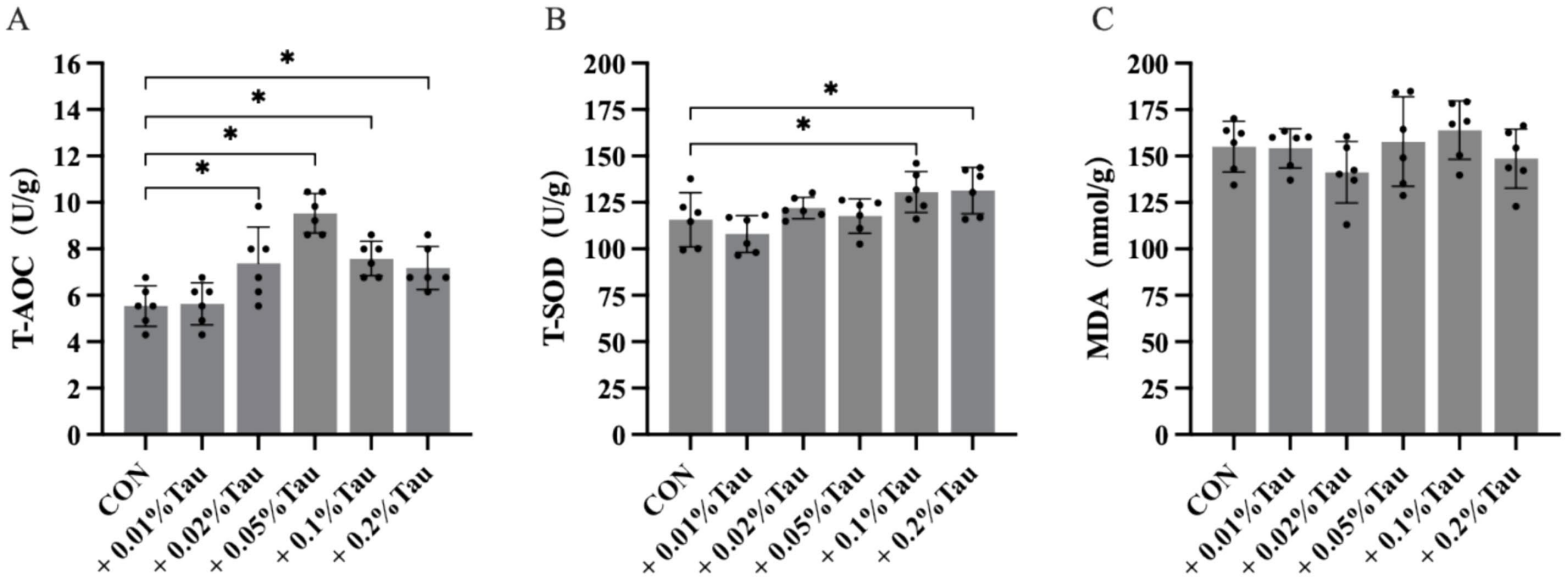
Effects of taurine on the antioxidant capacities of egg yolks. **(A)** T-AOC levels in egg yolks. **(B)** T-SOD levels in egg yolks. **(C)** MDA levels in egg yolks. The values represent the mean ± SD (*n* = 6), *p < 0.05 between the CON group and the other groups.

The addition of 0.02, 0.05, 0.1%, or 0.2% taurine to the hens’ diets significantly increased egg yolk T-AOC levels compared with those of the CON group (*p* < 0.05, Figure 2A); the egg yolk T-SOD levels of the 0.1 and 0.2% taurine-treated groups were significantly greater than those of the CON group (*p* < 0.05, Figure 2B). However, the egg yolk MDA contents was not significantly affected by taurine treatment in either the CON or treated groups (*p* > 0.05, Figure 2C).

### 3.5 Taurine increasing both saturated and unsaturated fatty acid contents in egg yolks

GC–MS revealed a total of 32 fatty acids in egg yolks, and the addition of 0.05, 0.1%, or 0.2% taurine to the hens’ diets significantly increased the saturated fatty acid (SFA) content of egg yolks compared with that of the CON group, with linear and quadratic trends following the increase in taurine content (*p* < 0.05, Table 5) and a significant increase in C15-0, C16-0, and C17-0 contents (*p* < 0.05). Adding 0.1% or 0.2% taurine to the diets significantly increased the C15-1 and C17-1 fatty acid contents of egg yolks (*p* < 0.05). It did not significantly increase the levels of total monounsaturated fatty acids (*p* > 0.05), but there was a linear and quadratic trend to increase with increasing taurine content (*p* < 0.05). The addition of taurine to diets significantly increased the PUFA contents of egg yolks, which increased both linearly and quadratically with increasing taurine content (*p* < 0.05). The addition of 0.05% or 0.1% taurine to the diet significantly increased the n-3 and n-6 PUFA contents of egg yolks (*p* < 0.05), and the addition of 0.2% taurine to the diet significantly increased the n-6 PUFA content (*p* < 0.05). Adding taurine to the diets significantly increased the C18-3n content of omega-3 fatty acids and the C18-2n content of omega-6 fatty acids in egg yolks, with a linear and quadratic increasing trend (*p* < 0.05) with increasing taurine content.

**Table 5:** Effects of taurine on fatty acid compositions in egg yolks.

Item	CON	+ 0.05% Tau	+ 0.1% Tau	+ 0.2% Tau	*p* value	Linear	Quadratic
Fatty acid composition (μg/g)							
Hexanoic acid (C6-0)	0.55 ± 0.08	0.69 ± 0.07	0.68 ± 0.13	0.66 ± 0.08	0.304	0.255	0.171
Nonanoic acid (C9-0)	1.55 ± 0.16	1.54 ± 0.24	2.19 ± 0.34	2.26 ± 0.80	0.161	**0.034**	0.118
n-Capric acid (C10-0)	1.71 ± 0.21	1.30 ± 0.03	1.94 ± 0.41	1.78 ± 0.06	0.049	0.321	0.510
Hexadecenoic acid (C11-0)	5.36 ± 0.18	5.34 ± 0.13	5.41 ± 0.8	5.47 ± 0.08	0.638	0.232	0.429
Lauric acid (C12-0)	6.64 ± 0.06	7.12 ± 0.09	7.16 ± 0.43	7.10 ± 0.10	0.071	0.055	**0.028**
Myristic acid (C14-0)	62.52 ± 7.90	78.28 ± 5.84	80.88 ± 11.58	84.07 ± 9.61	0.074	**0.015**	**0.030**
Pentadecanoic acid (C15-0)	27.05 ± 1.02	32.69 ± 1.25*	33.67 ± 0.75*	33.55 ± 1.56*	**< 0.001**	**0.002**	**< 0.001**
Palmitic acid (C16-0)	9241.45 ± 937.93	11003.65 ± 837.60*	11660.28 ± 1162.15*	11635.45 ± 522.35*	**0.033**	**0.009**	**0.010**
Margaric acid (C17-0)	75.40 ± 1.63	84.55 ± 3.64**	90.36 ± 2.03***	85.88 ± 1.53**	**< 0.001**	**0.008**	**< 0.001**
Stearic acid (C18-0)	4209.22 ± 552.80	4671.37 ± 378.07	5169.43 ± 383.26	5144.02 ± 224.85	0.057	**0.008**	**0.021**
Nonadecylic acid (C19-0)	62.35 ± 0.98	64.51 ± 1.91	64.81 ± 0.69	66.30 ± 1.84	0.060	**0.006**	**0.028**
Arachidic acid (C20-0)	58.85 ± 0.99	59.65 ± 1.75	60.57 ± 0.10	61.98 ± 1.52	0.073	**0.006**	**0.025**
Terdecanoic acid (C21-0)	49.11 ± 1.82	49.64 ± 1.55	49.63 ± 0.95	50.26 ± 0.78	0.766	0.272	0.554
behenic acid (C22-0)	51.23 ± 0.85	53.01 ± 1.54	52.73 ± 1.15	53.57 ± 0.77	0.145	**0.040**	0.106
Tricosanoic acid (C23-0)	24.28 ± 0.35	25.12 ± 0.40	24.74 ± 0.94	25.05 ± 0.36	0.327	0.232	0.380
Lignoceric acid (C24-0)	95.96 ± 2.38	97.47 ± 2.89	97.65 ± 0.69	98.91 ± 1.71	0.439	0.094	0.265
Myristoleic acid (C14-1)	25.42 ± 4.77	27.21 ± 0.83	26.83 ± 6.73	31.96 ± 5.50	0.449	0.139	0.297
10-Pentadecanoic acid (C15-1)	73.14 ± 5.32	86.49 ± 6.18	91.92 ± 1.91*	91.75 ± 11.98*	**0.042**	**0.010**	**0.013**
cis-9-Hexadecanoic acid (C16-1)	649.20 ± 103.39	822.16 ± 70.95	818.77 ± 208.56	961.54 ± 178.21	0.171	**0.028**	0.101
cis-11-Eicosenoic acid C20-1(cis-11)	94.55 ± 3.75	99.51 ± 4.07	105.95 ± 2.23**	107.16 ± 5.22**	**0.015**	**0.001**	**0.005**
trans-11-Eicosenoic acid (C20-1 T)	49.51 ± 0.01	50.24 ± 1.50	50.63 ± 0.60	52.03 ± 1.48	0.108	**0.012**	**0.044**
Oleic acid (C18-1n9c)	7702.72 ± 1178.42	8954.21 ± 728.21	9688.21 ± 1506.18	9777.63 ± 521.33	0.136	**0.022**	0.052
Elaidic acid (C18-1n9t)	530.56 ± 74.57	630.57 ± 74.96	680.90 ± 116.17	728.86 ± 96.74	0.130	**0.014**	0.050
Linoleic acid (C18-2n6)	3868.08 ± 471.06	4884.07 ± 500.32*	4839.50 ± 577.52*	5131.50 ± 75.38**	**0.038**	**0.013**	**0.023**
α-Linolenic acid (C18-3n3)	85.56 ± 4.43	101.12 ± 4.36*	102.96 ± 9.93**	101.09 ± 3.05*	**0.025**	**0.028**	**0.009**
γ-Linolenic acid (C18-3n6)	91.46 ± 2.75	105.10 ± 4.97**	99.82 ± 6.50	107.02 ± 2.73**	**0.012**	**0.021**	0.053
11,14-Eicosadienoic acid (C20-2)	98.39 ± 5.25	105.97 ± 8.75	108.68 ± 5.22	113.66 ± 7.01	0.116	**0.012**	**0.047**
cis 11,14,17-eicosotrienic acid	61.19 ± 2.32	61.49 ± 1.76	61.98 ± 1.42	63.03 ± 1.38	0.614	0.172	0.385
cis-8,11,14-Eicosatrienoic acid (C20-3n6)	173.37 ± 18.15	196.68 ± 5.55	198.66 ± 30.72	239.79 ± 24.78**	**0.034**	**0.005**	**0.018**
Arachidonic acid (C20-4n6)	1646.09 ± 269.35	1949.06 ± 136.53	1923.94 ± 230.59	2052.44 ± 32.85	0.135	**0.033**	0.086
cis-7,10,13,16-docosatetraenoic acid (C22-4(cis-7,10,13,16))	225.25 ± 24.73	253.79 ± 23.56	250.35 ± 28.63	254.04 ± 9.35	0.401	0.175	0.267
Cervonic acid (C22-6n3)	669.10 ± 120.12	851.64 ± 46.09	878.13 ± 94.24	804.49 ± 15.30	0.051	0.126	**0.016**
Saturated fatty acids (SFAs)	13973.26 ± 1482.44	16235.73 ± 1189.55	17402.14 ± 1553.30*	17356.30 ± 532.85*	**0.032**	**0.007**	**0.009**
Monounsaturated fatty acids (MUFAs)	9125.1 ± 1321.67	10670.37 ± 860.04	11463.20 ± 1837.25	11750.93 ± 774.11	0.127	**0.018**	**0.048**
Polyunsaturated fatty acids (PUFAs)	6918.49 ± 893.05	8508.92 ± 694.24*	8464.03 ± 954.81*	8867.07 ± 85.14*	**0.049**	**0.017**	**0.028**
*n*-6 PUFAs	5778.99 ± 746.62	7134.91 ± 623.90*	7061.92 ± 838.68*	7530.75 ± 84.77*	**0.047**	**0.013**	**0.030**
*n*-3 PUFAs	815.85 ± 122.22	1014.25 ± 46.60*	1043.07 ± 101.75*	968.62 ± 10.94	**0.041**	0.105	**0.013**
*n*-6/*n*-3 PUFAs	7.10 ± 0.33	7.03 ± 0.34	6.76 ± 0.29	7.78 ± 0.46*	**0.014**	0.149	**0.021**
Total unsaturated fatty acids	16043.59 ± 2098.58	19179.30 ± 1553.76	19927.23 ± 2791.72	20618.00 ± 850.54	0.089	**0.015**	**0.035**
Total fatty acids	30016.85 ± 3680.92	35415.03 ± 2742.04	37329.37 ± 4316.17	37974.31 ± 1375.36	0.059	**0.010**	**0.020**

The fatty acid content is based on the determination of the egg yolk dry matter; the values represent the mean ± SD (*n* = 3), *p < 0.05, **p < 0.01, and ***p < 0.001 between the CON group and the other groups. Bold values indicate statistically significant differences (*p* < 0.05) compared to the control group.

### 3.6 Taurine decreases serum and liver TG and TC contents by altering the expression of cholesterol metabolism-related genes in hens during the late laying period

**Figure 3:**
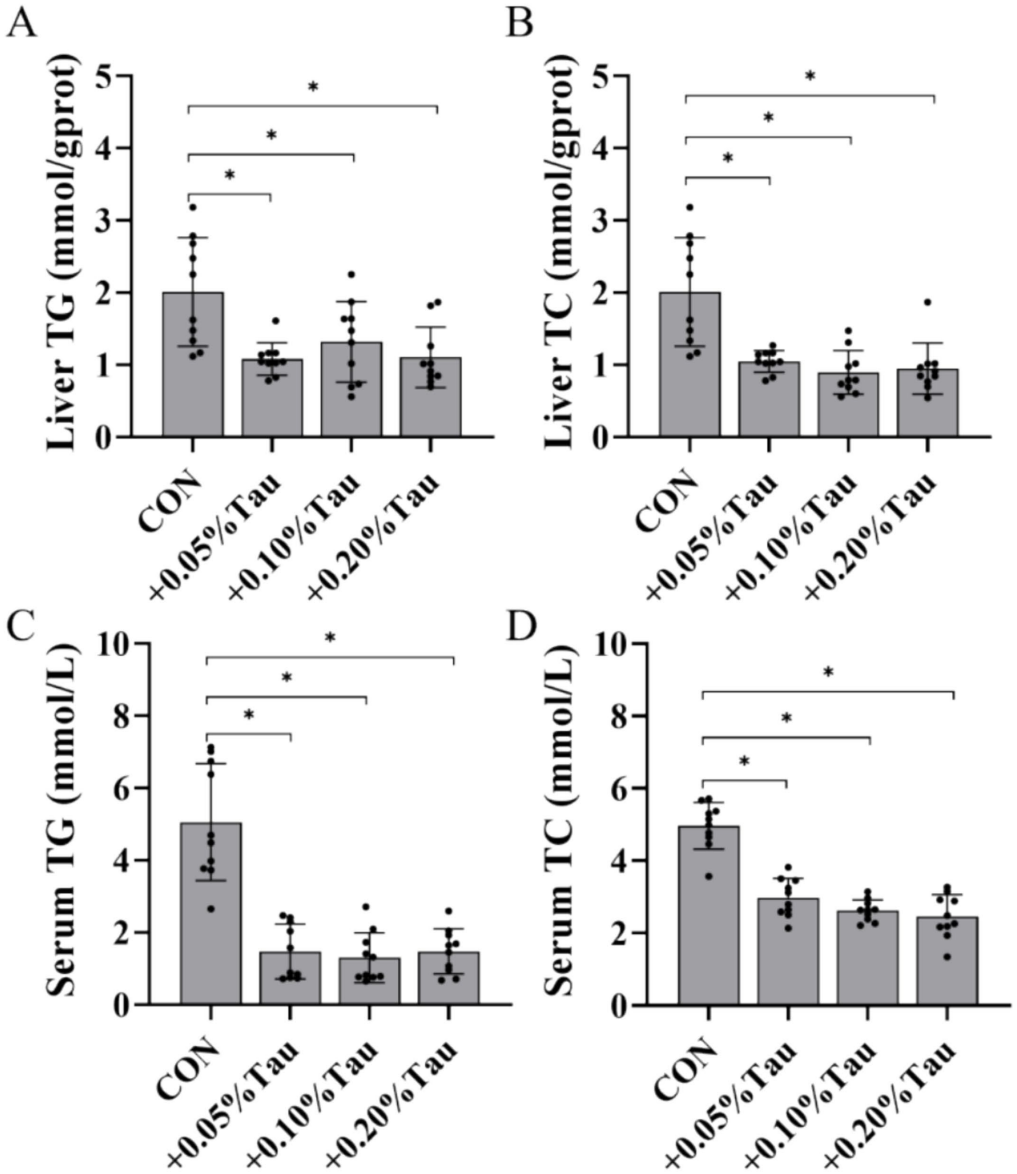
Effects of taurine on TG and TC levels in the liver and serum of laying hens during the late laying period. **(A)** TG levels in the liver. **(B)** TC levels in the liver. **(C)** Serum TG levels. **(D)** TC levels in the serum. TG, triglyceride; TC, total cholesterol. The values represent the mean ± SD (*n* = 10), *p < 0.05 between the CON group and the other groups.

**Figure 4:**
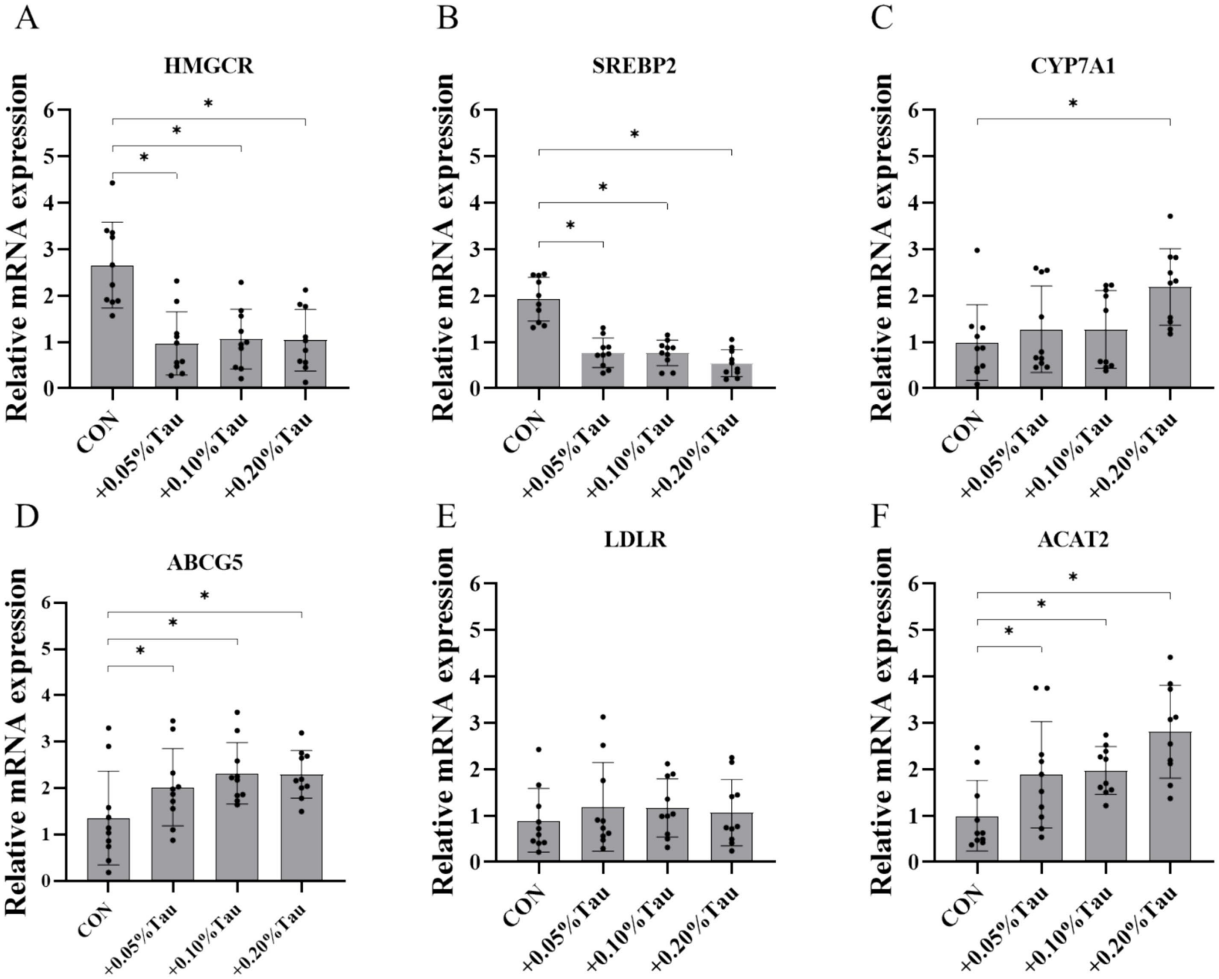
Effects of taurine on the expression of genes involved in cholesterol metabolism in the livers of laying hens. **(A)** HMGCR, recombinant 3-hydroxy-3-methylglutaryl coenzyme A reductase. **(B)** SREBP-2, sterol regulatory element binding protein-2. **(C)** CYP7A1, recombinant cytochrome P450 7A1. **(D)** ABCG5, ATP binding cassette transporter G5. **(E)** LDLR, recombinant low-density lipoprotein receptor **(F)** ACAT2, recombinant acetyl coenzyme A acetyltransferase 2. The values represent the mean ± SD (*n* = 10), *p < 0.05 between the CON group and the other groups.

The addition of 0.05, 0.1%, or 0.2% taurine to the diet significantly reduced the serum and liver TG and TC levels of aged laying hens during the late laying period compared with those in the CON group (*p* < 0.05; Figures 3A–D). The addition of 0.05, 0.1%, or 0.2% taurine to the diet significantly reduced the expression of *HMGC* and *SREBP-2* mRNA in the livers of aged laying hens (*p* < 0.05; Figures 4A,B) and increased the expression of *ABCG5* and *ACAT2* mRNA (*p* < 0.05; Figures 4D,F) but did not affect the expression of *LDLR* mRNA (*p* > 0.05; Figure 4E). Feeding a diet containing 0.2% taurine significantly increased the expression of liver *CYP7A1* mRNA in aged laying hens (*p* < 0.05; Figure 4C).

## 4 Discussion

In poultry production, the egg production rate and egg quality of laying hens gradually decreases at the late laying period of laying hens (4). Solving this production difficulty is a current challenge after antibiotic is prohibited.

In this study, dietary taurine supplementation significantly increased the average egg weight of laying hens in the late laying stage, and although no significant change in the laying rate was observed, there was a linear increase in egg production rate with increasing taurine dosage, with an average increase of about 4–5% compared to the CON group, and this has certain production significance in modern commercial poultry production. Taurine has antioxidant properties and immunomodulatory effects (40), potentially contributes to improves the occurrence of ovarian and oviduct damage and inflammation in laying hens, and increases the laying rate (14) and egg weight (18). However, while this suggests improved productivity in terms of egg mass, it does not unequivocally indicate overall enhanced performance.

As laying time increases, quality of eggs gradually decreases, including an increase in breakage rate, a decrease in Albumen height, lighter yolk color, and a decrease in storage time (41, 42). In this study, the addition of taurine to the hens’ diet reduced the yolk color, and as the laying hens’ weekly age increased, both the control and the taurine-treated groups presented a gradual decreasing trend in yolk color, which is consistent with the findings of previous studies (18). This reduction may result from taurine-altered lipid metabolism accelerating fatty acid incorporation, and competition with or interference in the transport and deposition of fat-soluble carotenoids, resulting in lighter yolk color (18), this requires further research. The Haugh unit and Albumen height are important indicators of egg quality. Notably, dietary taurine supplementation increased the Haugh units and Albumen heights of the eggs, indicate that feeding taurine-containing diets can improve egg quality.

Egg yolk contains a large amount of fatty substances, and improving the antioxidant capacity of eggs can prolong shelf-life (43). In this study, dietary supplementation of taurine increased the contents of SFAs and PUFAs, especially n-3 and n-6 PUFAs, in egg yolks. Previous studies from our research group found that dietary supplementation with 0.02% taurine can increase the content of PUFAs in serum (20), indicating that the increase in PUFAs content in egg yolks is somewhat associated with this. While elevated PUFAs offer nutritional benefits for human consumers, such as supporting cardiovascular and health (26–28), the concurrent rise in SFAs may offset these advantages, as high SFA intake is linked to adverse health outcomes (44).

Dietary taurine supplementation reduced the TG and TC contents in egg yolks and improved the antioxidant capacity of egg yolks, which was similar to the results of reducing the TC content of egg yolks by adding appropriate amounts of taurine to quail diets (45). This study revealed that taurine plays an important role in hepatic TC metabolism by reducing the mRNA expression of *HMGCR* and *SREBP2* in the liver, promoting the mRNA expression of the cholesterol transporter *ABCG5* and cholesterol esterase *ACAT2*, and enhancing the mRNA expression of *CYP7A1* to facilitate cholesterol conversion to bile acids, which in turn leads to a reduction in TC contents in serum, liver, and eggs, and these results are consistent with previous studies in birds (46, 47). Notably, *LDLR* mRNA expression remained unaffected, possibly due to avian hepatic cholesterol regulation prioritizing synthesis inhibition over receptor-mediated uptake.

## 5 Conclusion

In conclusion, supplementing 0.05 to 0.2% taurine in the diets of laying hens during the later stages of egg laying has a positive effect on egg quality, egg yolk unsaturated fatty acid levels and antioxidant capacity. Supplying taurine also effectively regulates TC metabolic pathways in the liver, which involve synthesis, conversion, excretion and esterification activities, thereby reducing the accumulation of TC in eggs. These findings provide a solid scientific basis for the application of taurine in poultry farming.

## Data Availability

The original contributions presented in the study are included in the article/supplementary material, further inquiries can be directed to the corresponding authors.
